# Temporal Evolution of Maternal Mortality: 1980-2019

**DOI:** 10.1055/s-0041-1735300

**Published:** 2021-10-20

**Authors:** Janete Vettorazzi, Edimárlei Gonsales Valério, Maria Alexandrina Zanatta, Mariana Hollmann Scheffler, Sergio Hofmeister de Almeida Martins Costa, José Geraldo Lopes Ramos

**Affiliations:** 1 Department of Gynecology and Obstetrics, Universidade Federal do Rio Grande do Sul, Porto Alegre, RS, Brazil; 2 Department of Gynecology and Obstetrics, Hospital de Clínicas de Porto Alegre, Porto Alegre, RS, Brazil

**Keywords:** maternal mortality, maternal mortality ratio, pregnancy-related death, severe maternal morbidity, pregnancy hypertension, acute fatty liver of pregnancy, postcesarean infection, septic abortion, mortalidade materna, razão de mortalidade materna, morte relacionada à gravidez, morbidade materna grave, hipertensão na gravidez, esteatose hepática aguda da gravidez, infecção pós-cesárea, aborto séptico

## Abstract

**Objective** To determine the profile of maternal deaths occurred in the period between 2000 and 2019 in the Hospital de Clínicas de Porto Alegre (HCPA, in the Portuguese acronym) and to compare it with maternal deaths between 1980 and 1999 in the same institution.

**Methods** Retrospective study that analyzed 2,481 medical records of women between 10 and 49 years old who died between 2000 and 2018. The present study was approved by the Ethics Committee (CAAE 78021417600005327).

**Results** After reviewing 2,481 medical records of women who died in reproductive age, 43 deaths had occurred during pregnancy or in the postpartum period. Of these, 28 were considered maternal deaths. The maternal mortality ratio was 37.6 per 100,000 live births. Regarding causes, 16 deaths (57.1%) were directly associated with pregnancy, 10 (35.1%) were indirectly associated, and 2 (7.1%) were unrelated. The main cause of death was hypertension during pregnancy (31.2%) followed by acute liver steatosis during pregnancy (25%). In the previous study, published in 2003 in the same institution ^4^ , the mortality rate was 129 per 100,000 live births, and most deaths were related to direct obstetric causes (62%). The main causes of death in this period were due to hypertensive complications (17.2%), followed by postcesarean infection (16%).

**Conclusion** Compared with data before the decade of 2000, there was an important reduction in maternal deaths due to infectious causes.

## Resumo

**Objetivo** Determinar o perfil dos óbitos maternos ocorridos no período de 2000 a 2019 no Hospital de Clínicas de Porto Alegre (HCPA) e comparar com os óbitos maternos entre 1980 e 1999 na mesma instituição.

**Métodos** Estudo retrospectivo que analisou 2.400 prontuários de mulheres entre 10 e 49 anos que morreram entre 2000 e 2019. O presente estudo foi aprovado pelo Comitê de Ética (CAAE 78021417600005327).

**Resultados** Após revisão de 2.481 prontuários de mulheres que morreram em idade reprodutiva, 43 mortes ocorreram durante a gravidez ou no período pós-parto. Destas, 28 foram considerados óbitos maternos. A taxa de mortalidade materna foi de 37.6 por 100.000 nascidos vivos. Em relação às causas, 16 óbitos (57.1%) estiveram diretamente associados à gravidez, 10 (35.1%) estiveram indiretamente associados e 2 (7.1%) não estiveram relacionados. A principal causa de morte foi hipertensão na gravidez (31.2%) seguida de esteatose hepática aguda da gravidez (25%). No estudo anterior, publicado em 2003 na mesma instituição ^4^ , a taxa de mortalidade foi de 129 por 100.000 nascidos vivos, e a maioria dos óbitos estava relacionada a causas obstétricas diretas (62%). As principais causas de óbito neste período foram por complicações hipertensivas (17.2%), seguidas de infecção pós-cesárea (16%).

**Conclusão** Em comparação com os dados anteriores à década de 2000, houve uma redução importante das mortes maternas por causas infecciosas.

## Introduction

Despite improvements in the last decades, maternal mortality remains a major global health problem. In 2017, nearly 810 women died every day from preventable causes related to pregnancy and childbirth in the world, and there is a considerable difference in rates between developed and developing countries. In the Americas, this disparity becomes more evident when we compare regions. The United States, for an example, had a maternal death rate of 17.4 per 100,000 live births in 2018, while in Brazil the rate was 60 per 100,000 live births in 2017. The lifetime risk of maternal death in Latin America and Caribe is 1/630, whereas the risk in North America is 1/ 3,100. 1 2

In the last report on maternal mortality of the World Health Organization (WHO), Brazil did not achieve the millennium goal, showing a 57.7% reduction in the maternal mortality ratio (MMR) in the past 25 years (104 per 100,000 live births in 1990; 66 per 100,000 live births in 2000; and 60 per 100,000 live births in 2017). However, Brazil is still considered a country with a very low maternal mortality rate (< 100 per 100,000 live births) according to the WHO classification. 1

In Brazil, there is a significant difficulty in correctly monitoring the level and the tendency of maternal mortality. This is mainly due to the lack of information and to the underreporting of death causes. Studies that map the epidemiology of maternal deaths are extremely important to outline strategies to combat this serious public health problem. 3

The present study aims to determine the profile of maternal deaths occurred in a tertiary care university hospital in southern Brazil from 2000 to 2019 and to compare it with the data obtained in the period from 1980 to 1999, which was analyzed in a previous study by Ramos et al. 4

## Methods

The present study adheres to the Strengthening the Reporting of Observational studies in Epidemiology (STROBE) guidelines for reporting observational studies. We performed a retrospective research analyzing all medical records of women between 10 and 49 years old who had died in the Hospital de Clínicas de Porto Alegre (HCPA, in the Portuguese acronym) from 2000 to 2019 and were pregnant or in the postpartum period (365 days after giving birth). Maternal death was defined as deaths that occurred during gestation or up to 42 days after birth that were due to related causes (direct) or were aggravated by gestation or by measures taken in relation to it (indirect), as recommended by the WHO.

Data were obtained through a query provided by the Information Technology Management Coordination (CGTI, in the Portuguese acronym) of the HCPA by applying selective filters such as age, deaths of pregnant women, number of births, and number of vaginal and cesarean deliveries, in the period of 2000–2019. After the selection of cases, the medical records of the patients were reviewed and the cause of death was analyzed. Individual data, such as comorbidities, were obtained. Later, the compilation of data was compared with a previous study conducted in the end of the last century in the same institution, from 1980 to 1999. This article was published in 2003 and is available in the references. Therefore, we considered period I from 1980 to 1999, and period II from 2000 to 2019. 4 5

Descriptive statistics are reported. Categorical variables are described as absolute (n) and relative (%) frequencies. Data was entered into Microsoft Excel 2013 (Microsoft Corporation, Redmond, WA, USA) spreadsheets for simple percentage analysis.

The present study was approved by the Ethics Committee in Research (CEP) of the HCPA under the Presentation Certificate for Ethical Appreciation (CAAE) number 78021417600005327.

## Results

We analyzed 2.481 medical records of women who died in reproductive age; of these, 43 were women in the pregnancy or postpartum period. Among these 43 deaths, 28 were considered maternal deaths. Considering that during the analyzed period there were 74,440 live births, the calculated maternal mortality ratio was of 37.6 deaths per 100,000 live births. When selecting only the births that had occurred in HCPA, 15 maternal deaths had occurred in the institution, resulting in a mortality ratio of 20.1 per 100,000 live births.

The mean age at death was 29.8 years old (standard deviation [SD] = 7 years), 65% of the women were white, 73% did not have a stable partner, and 85% had incomplete secondary education. Regarding the association between gestation and death, 16 deaths (57.1%) were directly associated to pregnancy, 10 (35.1%) were indirectly associated, and 2 (7.1%) were unrelated.

Table 1 describes the causes of maternal death in the HCPA between 2000 and 2019. The main cause of death directly related to gestation was pregnancy hypertension, with 5 cases (31.2%), followed by acute fatty liver of pregnancy, with 4 cases (25%). Infectious causes totaled 2 cases (12.5%) – 1 infected abortion and 1 necrotizing fasciitis of β-hemolytic streptococcus of Group A after vaginal delivery. No infectious cases were associated with cesarean sections. Puerperal hemorrhage and peripartum cardiomyopathy were associated with 2 cases (12.5%) each.

**Table 1:** Causes of Maternal Mortality at the Hospital de Clínicas de Porto Alegre between 2000 and 2019

**Direct Obstetric Causes**	***n***	**%**
Pregnancy hypertension	5	31.2
Fatty liver	4	25
Puerperal hemorrhage	2	12.5
Peripartum cardiomyopathy	2	12.5
Sepsis (necrotizing fasciitis)	1	6.2
Septic abortion	1	6.2
Amniotic embolism	1	6.2
Infection postcesarean section	0	0
Subtotal	16	100
**Indirect Obstetric Causes**	***n***	**%**
Cardiopathy	1	10
Pneumonia/ pneumopathies	2	20
Sepsis	2	20
AIDS	1	10
Neoplasm	1	10
Venous thromboembolism	0	0
Other causes*	3	30
Subtotal	10	100
Non-obstetric causes**	2	
Total deaths	28	

* Other indirect causes – hepatitis A, microangiopathy and idiopathic thrombocytopenic purpura.

** Unrelated causes – electric shock and unidentified (organ donation).

Among the indirect obstetric causes of maternal death, the most common were pneumopathy and sepsis (mainly for a respiratory reason, such as tuberculosis), each with 2 cases (20%). Cardiopathy (acute myocardial infarction), acquired immune deficiency syndrome and neoplasm accounted for 1 case each (10%). Unrelated deaths were due to electric shock and undetermined with organ donation. Considering maternal deaths only of the births that had occurred at the HCPA, the main causes remained pregnancy hypertension and fatty liver of pregnancy.

Among maternal deaths, there was a similar rate of vaginal delivery ( *n* = 10; 35.7%) and cesarean section ( *n* = 12; 42.8%). In contrast, 5 (17.8%) deaths occurred during pregnancy or abortion. One case had no data regarding this topic – the patient arrived directly at the intensive unit care after giving birth, and the mode of delivery was not recorded.

**Fig. 1:**
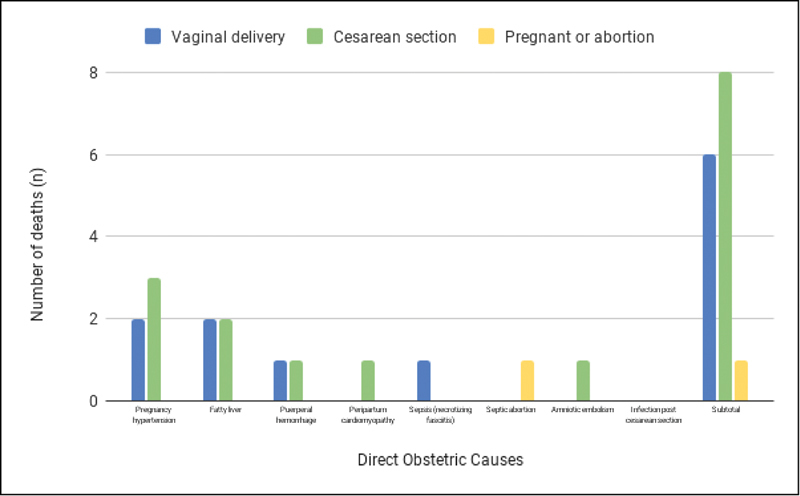
Delivery route and direct causes of maternal death.

The relation between direct causes of death and mode of delivery is shown in Figure 1 . The mortality ratio found was 1:4205 for vaginal delivery and 1:2261 for cesarean section (odds ratio [OR] = 0.37; 95% confidence interval [CI]: 0.13–1.09). The most common delivery mode was cesarean section (57%), compared with the vaginal route (42%). However, in the same period, the predominant way of delivery in the institution was the vaginal route, with a cesarean section rate of 31%.

**Fig. 2:**
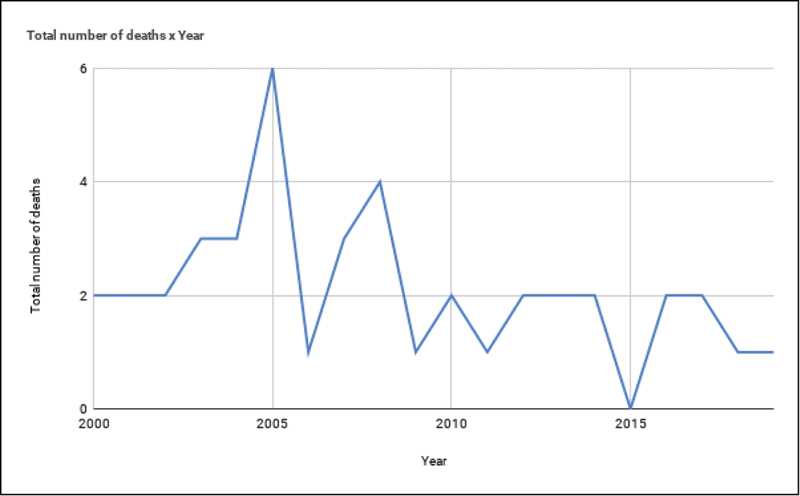
Maternal Mortality Rate between 2000 and 2019 in a Tertiary Care University Hospital of Southern Brazil.

Analysis by year including all deaths (direct, indirect, and unrelated) is shown in Figure 2 . There were no deaths in the year of 2015.

For comparison with the period from 1980 to 1999 in the same institution, 1,581 medical records were reviewed, of which 81 were considered maternal deaths. Ramos et al. 4 found a rate of 129 per 100,000 live births, and among those born in the HCPA, the rate was 90 per 100,000 live births. As for the characteristics of the studied population, 77.3% were white, 64.1% had a stable marital status, 58.5% attended at least 1 prenatal consultation, and 20.7% were primiparous. Death had occurred in pregnancy during prenatal care in 14 women (17.5%). The mode of delivery was cesarean section in 41 patients (50%) and vaginal in 15 (18.7%). The pregnancy ended in abortion in 10 cases (12.5%), and in 1 case (1.2%), it was possible to determine the gestational moment of death or the type of delivery.

**Fig. 3:**
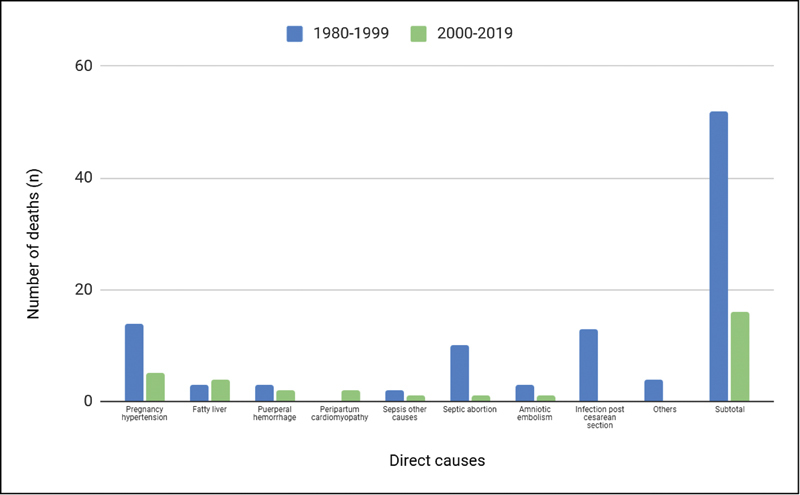
Comparison between direct causes of maternal death between the periods of 1980–1999 and 2000–2019.

**Fig. 4:**
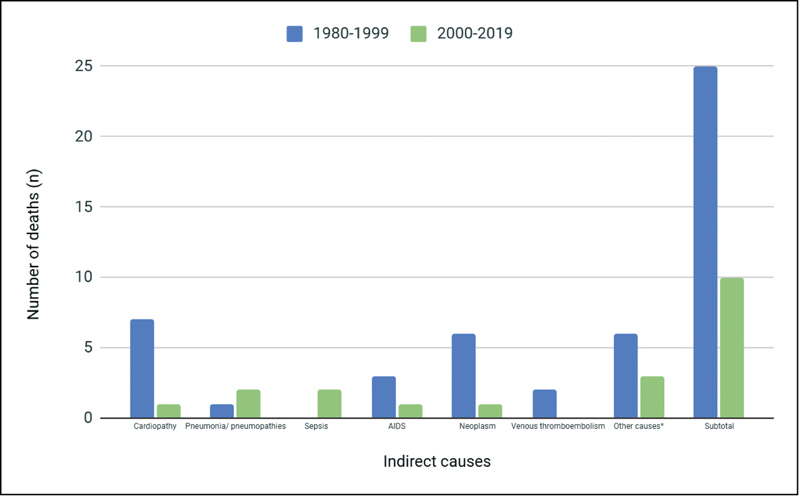
Comparison between indirect causes of maternal death between the periods of 1980–1999 and 2000–2019.

The same study by Ramos et al. 4 found that most deaths were related to direct obstetric causes, with 50 deaths (62%); and the main causes were hypertensive complications (14 cases; 17.2%), followed by postcesarean infection (13 cases; 16%), and septic abortion (10 cases; 12,3%). Heart disease was the predominant indirect cause of maternal death (8,6%). Figures 3 and 4 show a comparison of the different causes of maternal death in the two time periods mentioned.

## Discussion

According to the second report on maternal mortality published by the WHO in 2014, the main cause of maternal death in the world was postpartum hemorrhage (27%), followed by gestational hypertension and its complications (14%). In Brazil, however, the pattern is reversed – according to data analysis on 3 decades of maternal mortality in Brazil, performed by Morse et al., 6 the main cause of maternal death in Brazil is hypertensive complications. This seems to be the standard in all Latin America and was the second most common cause in our study. 6 7

The mortality ratio in the HCPA was 37.6 per 100,000 live births. Yet, if we consider only the births that occurred at the institution, the mortality ratio falls significantly to 20.1 per 100,000 live births. This rate is far below the national maternal mortality rate and is within the global mortality rate targets. It is safe to assume that this difference in rates can happen because, usually, the delivery assistance previously occurs in places with deficient care service; therefore, these patients arrive for attendance at the HCPA already in critical condition. In addition, it is possible to infer that the rate inherent only to our hospital births is better due to assistance based on established protocols, among other causes.

The causes of death in our study were similar to the national standard. In the HCPA, there is a predominance of direct causes of death, with pregnancy hypertension and acute fatty liver of pregnancy occupying the first and second place, respectively. One possible explanation for fatty liver in this position, despite being such a rare cause of maternal death nationally and worldwide, is the fact that the HCPA is a tertiary hospital and is the medical reference in the state of Rio Grande do Sul for cases requiring more complex care.

Comparing with the study previously performed in the institution in 2003, which evaluated maternal mortality in the HCPA between 1980 and 1999, a significant reduction in the maternal mortality rate was noticed. The comparison with rates found 19 years ago allows us to evaluate several issues. We can see a change in the population, with a subgroup of women currently getting pregnant later in life, and this may lead to a greater number and risk of comorbidities, such as pregnancy hypertension. As has also been noted, direct causes that are considered avoidable, like puerperal infections (especially after surgery), have dropped sharply. This is probably due to overall improvements in care. We also found a drop in cesarean rates. Moreover, we observed that complications associated with hypertension remain an important cause of death, with rates increasing in comparison with previous years (31.2% in our study versus 17% found by Ramos et al.). 4 One explanation is that pregnant women are currently presenting more risk factors for this gestational comorbidity, such as obesity and metabolic syndrome.

In women that had already given birth, maternal deaths associated with cesarean section represented approximately twice the number of deaths related to vaginal delivery. However, when we analyzed the causes of death related to the delivery route, we did not find any causes directly related to the surgical act, except for one case of anesthetic complications. This difference in risk may, therefore, mean that the cases that are taken to the cesarean section are more serious and tend to have worse outcomes, and not necessarily that the surgical procedure implies a higher risk of death.

We noticed a significant improvement in the maternal mortality rate at the HCPA, with an imperative decrease in that rate over the past 19 years. In addition to that, changes in the etiological pattern of deaths, such as the reduction in infection and hemorrhage causes, represents an improvement in healthcare. Aligned with strategies for reducing national and worldwide maternal mortality, this reflects in a drop in direct and avoidable obstetric causes of death.

We are still far from achieving acceptable maternal mortality rates. The struggle against maternal mortality is now on the agenda of the Sustainable Development Objectives (SDGs) that have replaced the Millennium Development Goals (MDGs) since 2015. This is a worldwide effort to eliminate preventable maternal mortality in the years 2016 to 2030. One of its goals defined by sustainable development goals (SDG) is to reduce the overall ratio to < 70 maternal deaths per 100,000 live births (SDG 3.1). The global maternal death rate is currently ∼ 210 deaths per 100,000 live births. In Brazil, the goal is to reduce mortality to 30 deaths per 100,000 live births. 8 9

From 2000 to 2017, the global maternal mortality ratio declined by 38% – from 342 deaths to 211 deaths per 100,000 live births according to the United Nations (UN) inter-agency estimates. This means an annual reduction of 2.9 per cent, which is less than half of the 6.4 per cent annual rate needed to achieve the Sustainable Development global goal of 70 maternal deaths per 100,000 live births. In the HCPA, in a period of 20 years, the mortality reduction rate was 70%, meaning a 3.5% reduction per year. 1

Our study has some limitations. One of them is the long-term retrospective research method with analysis of medical records, part of which was not electronically registered – only in early 2005, HCPA began using digitalized medical records. Therefore, some information could have been lost during data collection. Another issue is the change in some classifications of maternal death in relation to the comparative study of records from 1980 to 1999.

## Conclusion

Maternal mortality is still an important matter that must be analyzed and discussed broadly, since it occurs in unacceptable numbers around the world and in our country. Therefore, studies like ours, with the aim at mapping the main conditions that lead to the death of these women, are one of the strategies that try to change this reality. In this context, we highlight that the lower risk of maternal death in our institution can be associated with assistance based on protocols of care in the physiological process of delivery.
